# Trifloxystrobin-triggered Drp1 hyperactivation biases mitophagy and imposes long-lasting SVCV susceptibility in teleost

**DOI:** 10.1128/jvi.00445-26

**Published:** 2026-06-01

**Authors:** Huan Wang, Yang Hu, Lei Liu, Jiong Chen

**Affiliations:** 1 State Key Laboratory for Quality and Safety of Agro-Products, Ningbo University 47862, Ningbo, China; 2 Laboratory of Biochemistry and Molecular Biology, School of Marine Sciences, Ningbo University 47862, Ningbo, China; 3 Key Laboratory of Aquacultural Biotechnology of Ministry of Education, Ningbo University 47862, Ningbo, China; University of Kentucky College of Medicine, Lexington, Kentucky, USA

**Keywords:** strobilurin fungicides, mitophagy, antiviral, SVCV, mitochondrial dynamics, virus susceptibility

## Abstract

Environmental pollutants are increasingly recognized as disease modifiers, reshaping host homeostasis and shifting host-pathogen dynamics toward higher infection risk in aquatic ecosystems. Here, we show that the widely used strobilurin fungicide trifloxystrobin (TFS) persistently erodes antiviral competence in fish and increases susceptibility to spring viremia of carp virus (SVCV) by driving dynamin-related protein 1 (Drp1)-mediated excessive mitophagy and sustained mitochondrial dysfunction. Using epithelioma papulosum cyprini (EPC) cells and zebrafish as complementary models, we find that environmentally plausible TFS exposures (2.5–25 μg/L) elevate SVCV permissiveness; notably, this phenotype resolves incompletely after chemical withdrawal. Transcriptomics revealed a dose-concordant shift toward stress/innate immune signaling and mitophagy programs, alongside broad repression of proliferative and DNA-repair pathways. Consistently, TFS induces persistent mitochondrial membrane depolarization, promotes fragmentation and ultrastructural deterioration, and increases mitochondria–lysosome coupling. Mechanistically, TFS elevates Drp1 abundance and Ser616 phosphorylation, promotes Drp1 recruitment to mitochondria, and sustains microtubule-associated protein 1 light chain 3B (LC3B)/lysosomal-associated membrane protein 2 (LAMP2) engagement, accompanied by persistent induction of core autophagy regulators (*gabarap*, *atg5*, *wipi1*, and *ambra1*) across extended recovery windows. *In vivo*, prolonged TFS exposure similarly yields durable enhancement of SVCV susceptibility even after long recovery periods, indicating incomplete restoration of host resistance. Together, these findings link a major agricultural fungicide to persistent Drp1-driven mitophagy overactivation and identify long-term antiviral resistance as an ecologically relevant endpoint for pesticide risk assessment and aquatic disease forecasting.

**IMPORTANCE:**

Viral diseases pose a significant challenge to sustainable aquaculture, and effective antiviral interventions remain limited. In this study, we reveal that trifloxystrobin, a widely used fungicide, induces mitochondrial dysfunction and Drp1-mediated excessive mitophagy, leading to long-term suppression of antiviral immune responses in fish. Importantly, this work identifies mitochondrial dynamics as a key determinant of viral susceptibility and demonstrates how environmental pollutants can reshape host-pathogen interactions. By linking mitophagy and Drp1 activation to increased spring viremia of carp virus susceptibility, our findings provide a novel perspective on how pollutants may exacerbate viral infections in aquaculture species. This work underscores the urgent need for ecosystem-based antiviral strategies and offers a mechanistic framework for assessing ecological risks posed by common agricultural chemicals, thereby informing environmental and disease management in aquaculture.

## INTRODUCTION

Aquatic ecosystems are on the frontline of the One Health paradigm, acting simultaneously as sinks for anthropogenic chemicals and as arenas where infectious agents persist, evolve, and spill over across species. This convergence is intensifying as surface waters increasingly carry complex, low-concentration mixtures of agricultural pesticides, veterinary pharmaceuticals, and other industrial pollutants that can accumulate across seasons and life stages, generating chronic low-level background exposure rather than discrete toxic events (1–3). Importantly, such exposures do not need to cause overt lethality to reshape disease risk: by rewiring immunometabolic set points, weakening barrier defenses, and perturbing host-associated microbiota, chemical stressors can erode the immune barrier that normally constrains pathogen amplification in the water column and within susceptible hosts (4, 5). Consistent with this concept, contaminant exposure has repeatedly been associated with impaired immune function and heightened susceptibility to infection in fish and other aquatic organisms (6), yet immunotoxicity and infection outcomes remain insufficiently integrated into routine environmental risk assessment, leaving a major gap in our ability to forecast outbreak risk under realistic multi-stressor conditions (4, 7).

Strobilurin (QoI) fungicides are among the best-selling modern fungicide classes, owing to broad-spectrum efficacy and a distinctive mode of action that inhibits mitochondrial respiration at the cytochrome bc1 complex (8, 9). Their intensive use has resulted in pervasive aquatic exposure via runoff/leaching, spray drift, and wastewater inputs, while compound-specific persistence supports chronic, low-dose occurrence in surface waters and sediments (9–13). Accordingly, strobilurins are increasingly detected across aquatic compartments, including riverine systems and drinking-water supply chains, and evidence of tissue residues/bioconcentration in fish raises concerns about trophic transfer through aquatic food webs (10, 12, 14–18). At environmentally relevant concentrations, representative strobilurins can cause lethal and sublethal outcomes in aquatic organisms, including malformations and developmental/reproductive impairment (19, 20). Notably, beyond these classical endpoints, emerging studies report immunotoxic effects across taxa, such as reprogrammed immune gene expression in fish and suppressed macrophage/lymphocyte functions in mammals, yet the mechanisms that connect realistic exposure to durable immune dysfunction remain poorly defined (21, 22). Given that mitochondria integrate aerobic respiration and redox homeostasis, and that strobilurins disrupt oxidative phosphorylation (OXPHOS) and bioenergetics, mitochondria have been proposed as a primary toxicity node in fish; however, how mitochondria-initiated dysfunction is coupled to impaired immune defense programs, potentially via dysregulated mitochondrial quality control, remains largely unresolved (8, 23, 24).

Mitophagy is a selective autophagy pathway that removes damaged or excess mitochondria via lysosomal degradation to maintain mitochondrial quality control (25, 26). Although basal mitophagy is homeostatic, excessive or pathogen-hijacked mitophagy can suppress antiviral innate immunity by depleting mitochondria and destabilizing mitochondria-anchored signaling hubs (27). Notably, mitochondrial antiviral signaling protein (MAVS) is anchored on the mitochondrial outer membrane, and enhanced autophagy/mitophagy has been shown to promote MAVS turnover, thereby weakening downstream signaling and blunting type I interferon and interferon-stimulated gene (ISG) induction (27–29). In environmental toxicology, this axis is particularly relevant because mitochondrial toxicants can engage stress-responsive (mito)autophagy. Indeed, strobilurins have been reported to trigger mitochondria-directed clearance pathways across models, including mitophagy in human cells and AMP-activated protein kinase (AMPK)–mechanistic target of rapamycin (mTOR)/reactive oxygen species (ROS)-linked (auto)mitophagy in macrophages, implicating a direct interface with immune cell physiology (30, 31). Consistent with these mechanistic observations, ecotoxicological studies further suggest that chronic strobilurin exposure can perturb mitochondrial energy balance and dynamics and is accompanied by impaired antiviral gene programs and heightened susceptibility to rhabdovirus infection and transmission in fish (32–34). Together, these findings support a unifying hypothesis that strobilurins promote antiviral vulnerability by driving mitochondrial fragmentation and excessive mitophagy.

Spring viremia of carp virus (SVCV), a non-segmented negative-sense ssRNA rhabdovirus, is the causative agent of spring viremia of carp and is listed by the World Organisation for Animal Health (WOAH) because of its substantial threat to aquaculture and aquatic ecosystems. Leveraging zebrafish (*Danio rerio*) and the cyprinid epithelioma papulosum cyprini (EPC) cell line as complementary *in vivo* and *in vitro* models, we asked whether environmentally relevant, long-term exposure to trifloxystrobin (TFS) produces persistent defects in antiviral resistance. Specifically, we quantified SVCV susceptibility after prolonged exposure and during extended recovery, evaluated the persistence and reversibility of TFS-induced autophagy/antiviral suppression, and profiled mitochondrial dysfunction spanning bioenergetics, membrane potential, ultrastructure, and mitochondria–lysosome coupling. Finally, we tested the mechanistic contribution of dynamin-related protein 1 (Drp1)-dependent mitochondrial fission and sustained mitophagy to TFS-enhanced SVCV susceptibility. By explicitly modeling exposure and recovery, our work provides mechanistic insight into how a widely used fungicide can chronically reprogram mitochondrial quality control and elevate the risk of viral disease in fish.

## RESULTS

### Persistent, exposure-duration–dependent antiviral suppression after TFS withdrawal

**Fig 1:**
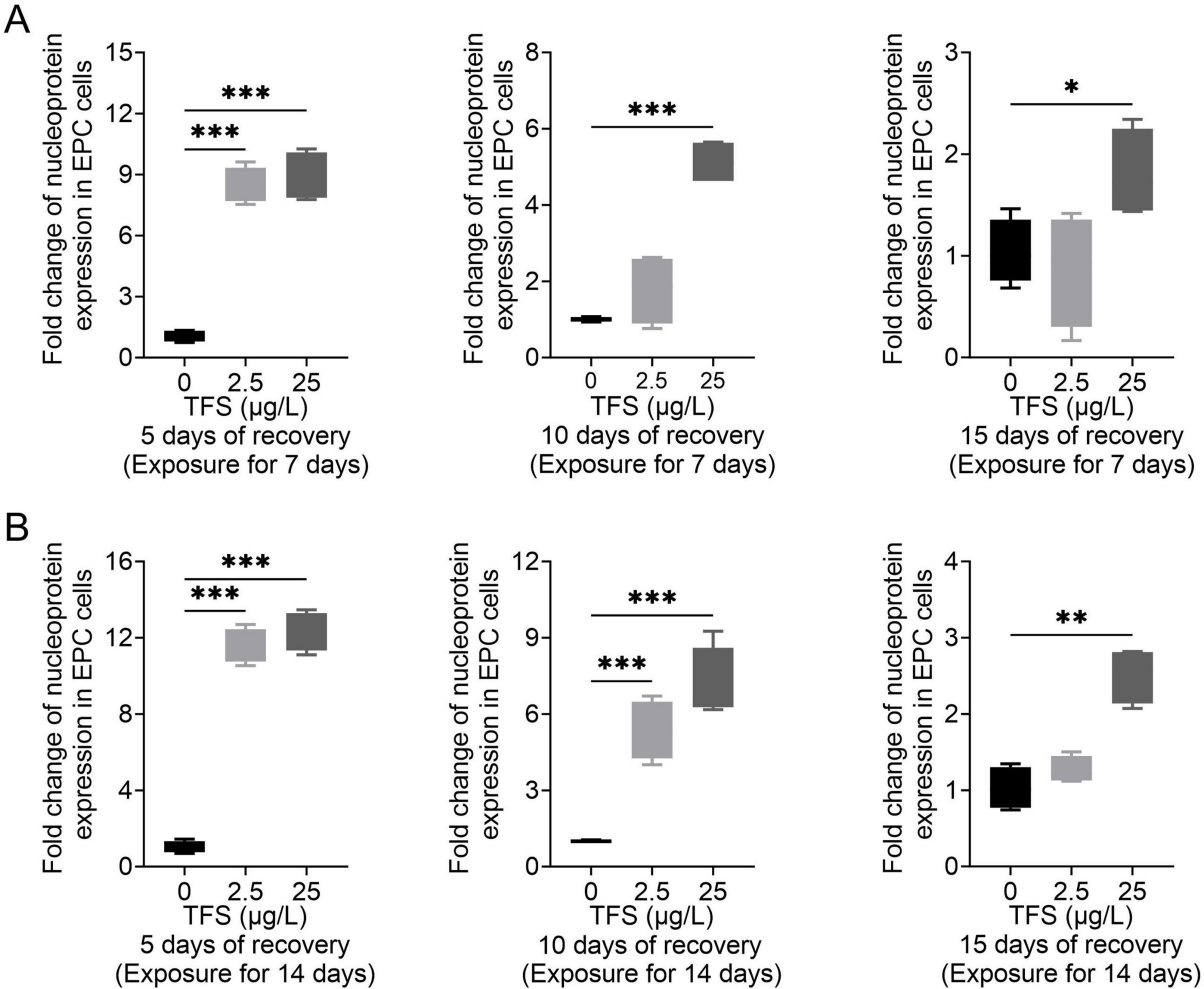
Prolonged TFS exposure produces an incompletely reversible increase in SVCV susceptibility in EPC cells. EPC cells were exposed to TFS (2.5 or 25 μg/L) for 7 d (**A**) or 14 d (**B**), transferred to TFS-free medium for 5-, 10-, or 15-d recovery, and then challenged with SVCV. Viral susceptibility was quantified by SVCV nucleoprotein expression (fold change relative to the control). Longer TFS exposures resulted in a durable elevation of nucleoprotein expression that persisted throughout the recovery window, with the 25 μg/L group remaining significantly above baseline at later recovery time points. Mean ± SD; two-tailed unpaired Student’s *t* test; *P ≤ 0.05, **P ≤ 0.01, ***P ≤ 0.001.

To test whether TFS-induced antiviral suppression persists after chemical withdrawal, EPC cells were exposed to TFS (2.5 or 25 μg/L) for 3, 7, or 14 d, allowed to recover for 5–15 d, and then challenged with SVCV; infection was quantified by SVCV nucleoprotein (N) expression. Following a brief 3-d exposure, susceptibility was transiently elevated after 5-d recovery but fully normalized by 10-d recovery (Fig. S1). In contrast, longer exposures produced a durable phenotype. After 7-d exposure, N expression remained markedly increased after 5-d recovery and, although it declined with time, it was still significantly higher than control at 10- to 15-d recovery, with the 25 μg/L group maintaining an ~2-fold elevation at 15 d (Fig. 1A). Extending exposure to 14 d further strengthened persistence: N expression stayed robustly elevated after 5- to 10-d recovery and remained significantly above baseline at 15-d recovery in the 25 μg/L group (Fig. 1B). Collectively, these data indicate that recovery of antiviral capacity is incomplete and strongly dependent on exposure duration, with prolonged TFS exposure imposing a long-lasting increase in SVCV permissiveness. *In vivo*, zebrafish exposed to TFS for 7 or 14 d recovered to near-control levels of SVCV N gene expression after 30 d (Fig. S2). However, longer exposures produced sustained susceptibility: 28-d exposure resulted in significantly elevated N expression even after 65-d recovery (>20-fold, *P* < 0.001), and fish exposed for 56 d remained more susceptible than controls after 95-d recovery (Fig. S3). Consistent with these phenotypes, recovery of interferon-related antiviral gene expression also showed clear exposure-duration dependence in both EPC cells and zebrafish. Following short-term exposure, including 3-d exposure in EPC cells with 10-d recovery and 7-d exposure in zebrafish with 15-d recovery, *ifn1*, *viperin*, *mx1*, and *isg15* expression largely returned to near-control levels. In contrast, after prolonged exposure, including 14-d exposure in EPC cells with 15-d recovery and 56-d exposure in zebrafish with 95-d recovery, these genes remained significantly suppressed (Fig. S4 and S5). Together, these results demonstrate that long-term TFS exposure can cause persistent, only partially reversible impairment of antiviral resistance, consistent across cellular and organismal models.

### TFS drives a mitophagy-biased stress program with broad repression of proliferative/repair pathways

**Fig 2:**
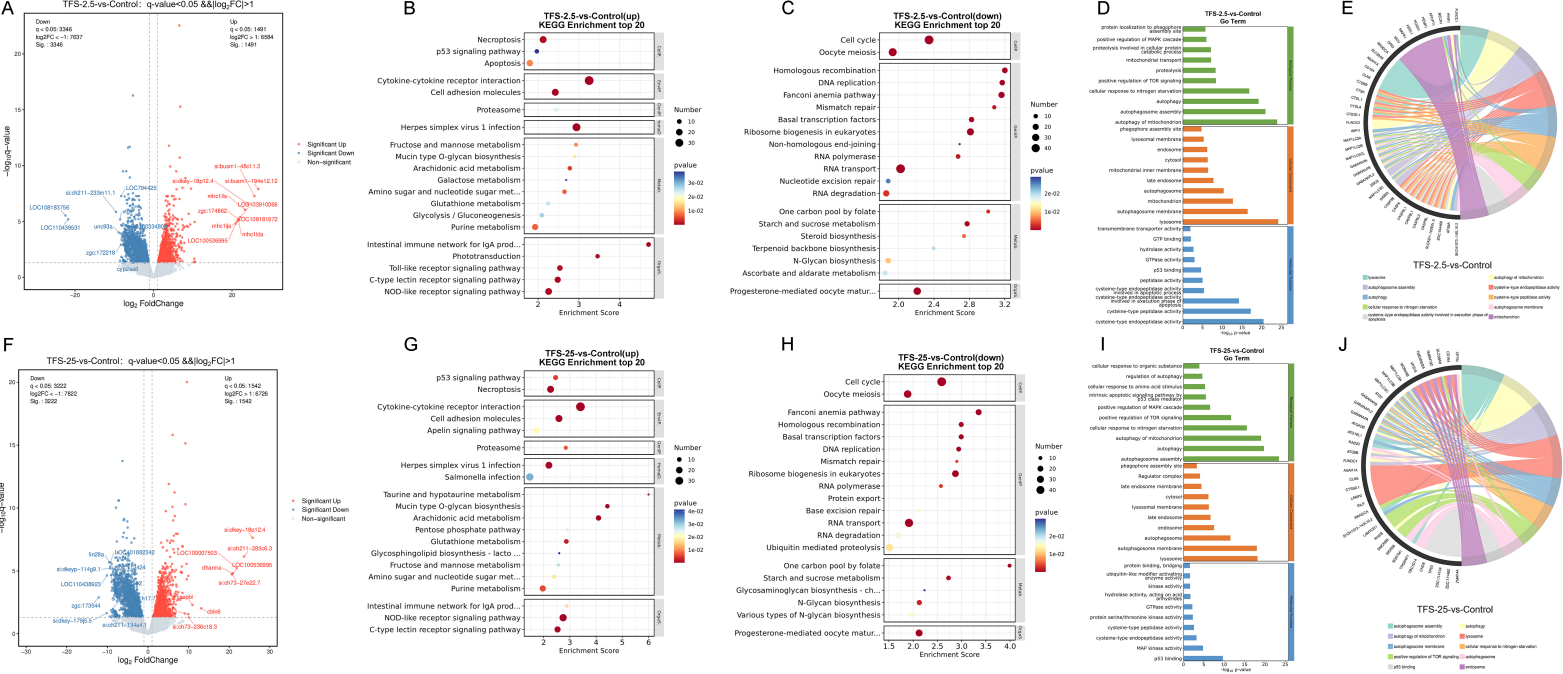
RNA-seq reveals dose-concordant activation of stress/innate immune pathways and mitophagy programs under TFS exposure. (**A and F**) Volcano plots of DEGs in the TFS 2.5-vs-Control and TFS 25-vs-Control comparisons; red, significantly upregulated; blue, significantly downregulated; gray, non-significant. (**B and G**) KEGG pathway enrichment analysis of upregulated DEGs in the TFS 2.5-vs-Control and TFS 25-vs-Control comparisons. Dot size indicates the number of DEGs per pathway, and dot color represents enrichment significance as raw P values (P < 0.05). (**C and H**) KEGG pathway enrichment analysis of downregulated DEGs in the TFS 2.5-vs-Control and TFS 25-vs-Control comparisons. Dot size indicates the number of DEGs per pathway, and dot color represents enrichment significance as raw P values. (**D and I**) GO term enrichment analysis of DEGs in the TFS 2.5-vs-Control and TFS 25-vs-Control comparisons, grouped by biological process, cellular component, and molecular function. (**E and J**) Chord diagrams linking representative enriched GO terms with associated genes in the TFS 2.5-vs-Control and TFS 25-vs-Control comparisons, highlighting autophagy/mitophagy-related gene modules.

RNA-seq of EPC cells exposed to TFS (2.5 or 25 μg/L) for 14 d revealed extensive, dose-concordant remodeling of the transcriptome (Fig. 2A), with a dominant downregulation signature (2.5 μg/L: 1,491 up/3,346 down; 25 μg/L: 1,542 up/3,222 down). Kyoto Encyclopedia of Genes and Genomes (KEGG) analyses showed that upregulated differentially expressed genes (DEGs) were enriched in stress and innate immune pathways, prominently p53 signaling, apoptosis/necroptosis, cytokine–cytokine receptor interaction, and pattern recognition receptor (PRR)-related programs (e.g., NOD-like receptor and C-type lectin receptor signaling) (Fig. 2B). Conversely, downregulated DEGs were enriched for cell cycle, DNA replication, and DNA repair/recombination modules, consistent with suppression of proliferative and macromolecular maintenance capacity (Fig. 2C). Gene Ontology (GO) enrichment highlighted robust activation of autophagy/mitophagy-related terms (including mitophagy, autophagosome membrane/assembly, and phagophore assembly site) together with stress-signaling terms (Fig. 2D). Chord mapping identified a shared set of autophagy core regulators, including gabarap, atg5, wipi1, and ambra1, as recurrent hubs linking multiple mitophagy/autophagosome and lysosome-associated processes at both doses (Fig. 2E). Collectively, these data define a TFS-induced transcriptional state characterized by mitochondria-stress–linked, mitophagy-biased activation coupled to broad repression of proliferation/repair programs.

### TFS induces persistent mitochondrial depolarization and fragmentation that incompletely recovers after withdrawal

**Fig 3:**
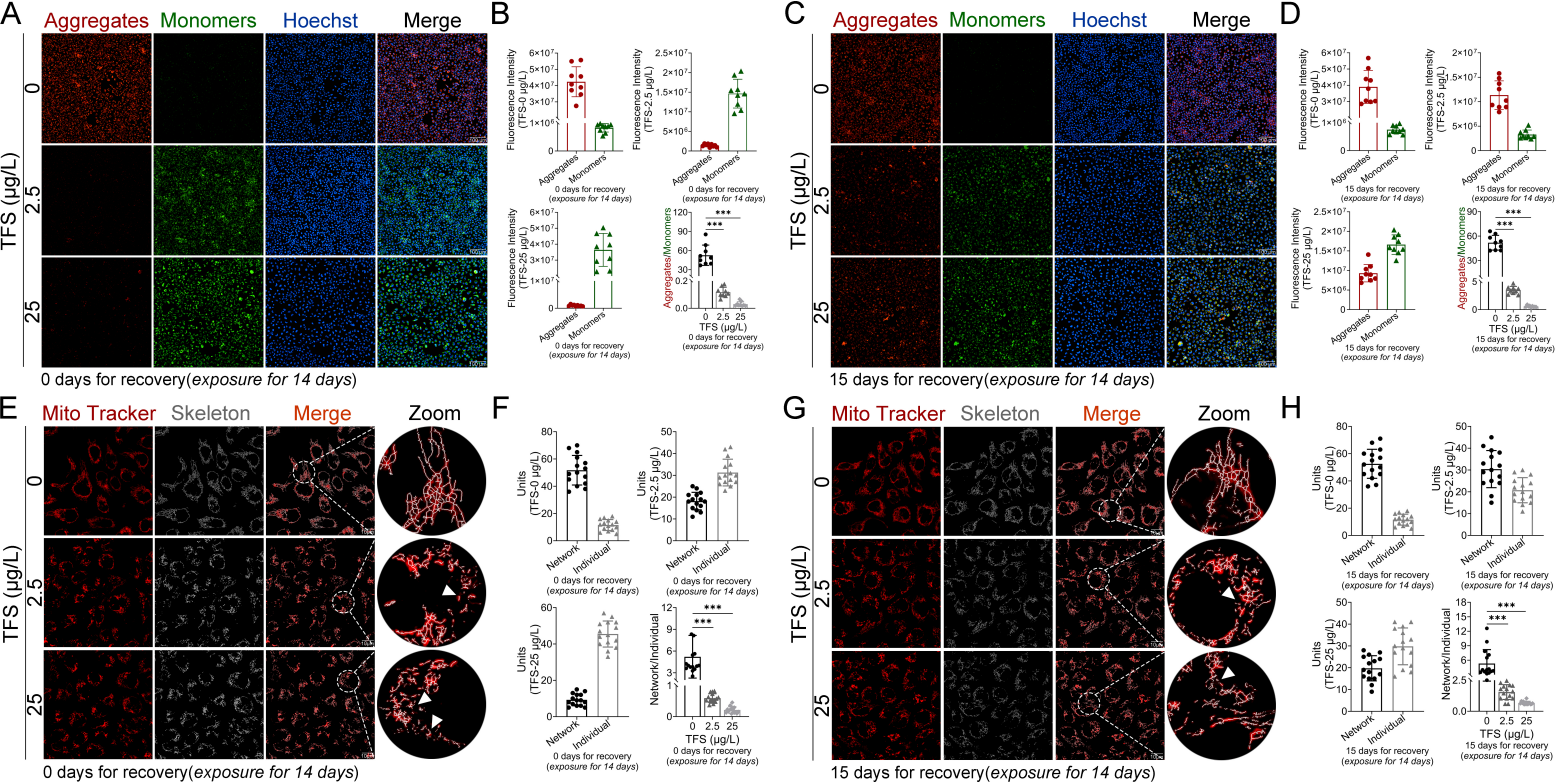
TFS causes Δψm depolarization and mitochondrial fragmentation with incomplete recovery. (**A and C**) Representative JC-1 images showing red aggregates and green monomers with Hoechst nuclear staining (blue) at 0-d recovery and 15-d recovery. (**B and D**) Quantification of JC-1 aggregate and monomer fluorescence intensity and the aggregate/monomer ratio at 0-d recovery and 15-d recovery. (**E and G**) Representative mitochondrial morphology images stained with MitoTracker (red), corresponding MiNA skeletonization (gray), merged views, and magnified regions (zoom) at 0-d recovery and 15-d recovery. (**F and H**) MiNA-based quantification of mitochondrial morphology, including networked and individual/fragmented mitochondrial units and the network/individual ratio at 0-d recovery and 15-d recovery. Data are expressed as mean ± SD, and a two-tailed unpaired Student’s *t* test is used. *P ≤ 0.05, **P ≤ 0.01, and ***P ≤ 0.001.

To determine whether TFS causes sustained mitochondrial dysfunction, we assessed mitochondrial membrane potential (Δψm) and mitochondrial network integrity immediately after exposure and following recovery. Using JC-1 staining, control EPC cells displayed strong red JC-1 aggregates with minimal green monomers, consistent with intact Δψm. In contrast, after 14 d TFS exposure, red aggregate fluorescence decreased while green monomer fluorescence increased in a concentration-dependent manner, resulting in a marked reduction in the aggregate/monomer ratio, indicating dose-dependent Δψm depolarization (Fig. 3A and B). After transferring exposed cells to TFS-free medium for 15 d, Δψm showed partial recovery, evidenced by increased aggregate signal and reduced monomer signal compared with the 0-d recovery group; however, the aggregate/monomer ratio remained significantly lower than control, demonstrating incomplete restoration of mitochondrial function (Fig. 3C and D). Consistent with these functional defects, TFS also disrupted mitochondrial architecture. MitoTracker staining revealed that control cells maintained a highly interconnected tubular network, whereas 14-d TFS exposure caused pronounced mitochondrial fragmentation, with increased isolated, punctate structures (Fig. 3E). Quantification using the Mitochondrial Network Analysis (MiNA) of ImageJ plugin confirmed a dose-dependent reduction in networked mitochondria and a concomitant increase in fragmented/individual units, leading to a decreased network-to-individual ratio (Fig. 3F). Notably, mitochondrial morphology recovered more slowly than expected after chemical withdrawal: even following 15-d recovery, the mitochondrial network remained incompletely reconstituted, and fragmentation-associated metrics were still significantly different from controls (Fig. 3G and H). Together, these results show that TFS induces persistent Δψm depolarization and mitochondrial fragmentation, and that both functional and structural impairments are only partially reversible after exposure cessation.

### TFS causes persistent mitochondrial ultrastructural damage and sustained mitochondria–lysosome coupling

**Fig 4:**
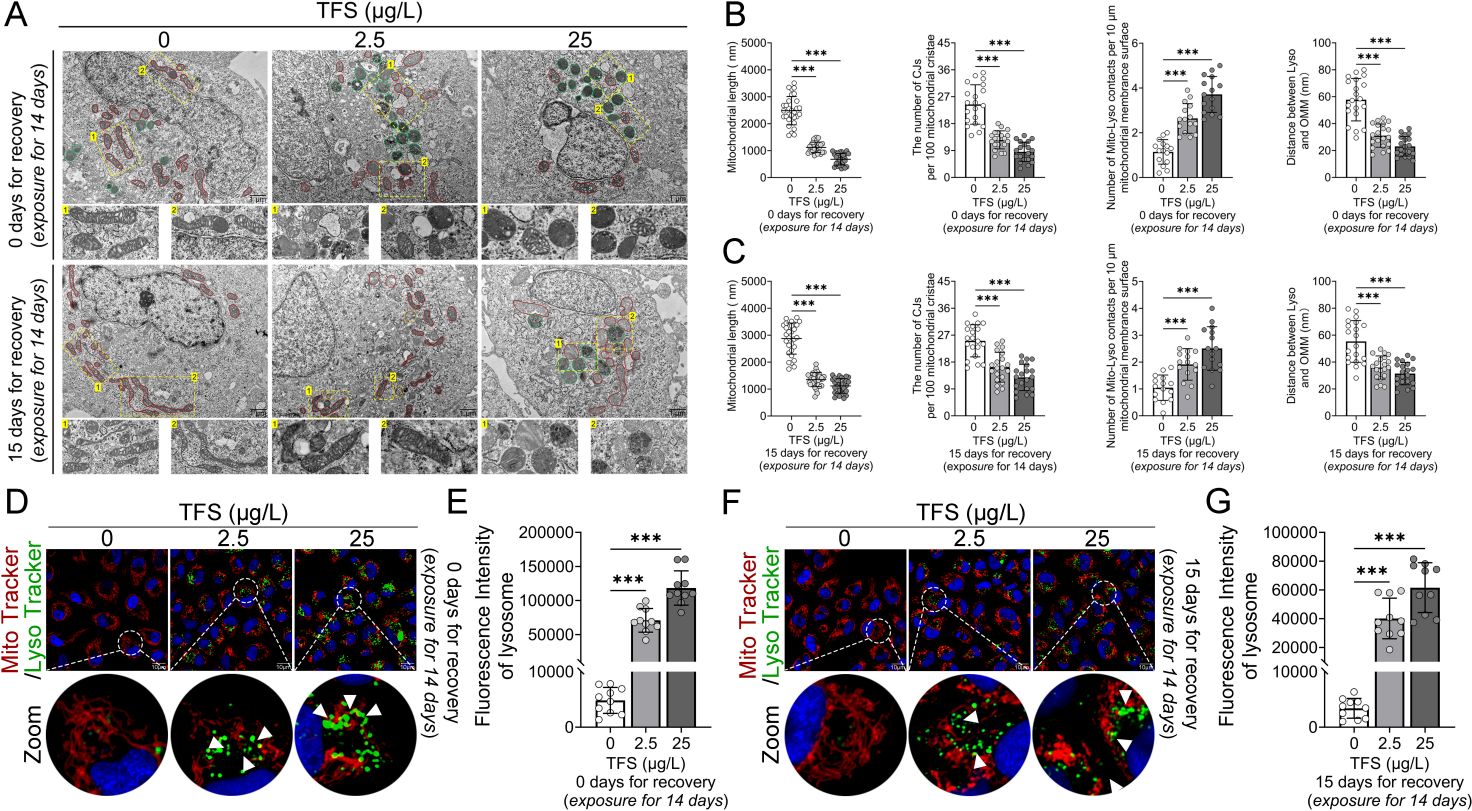
TFS causes persistent mitochondrial ultrastructural damage and sustained mitochondria–lysosome coupling. (**A**) Representative TEM images showing mitochondrial ultrastructure and mitochondria–lysosome interactions at 0-d and 15-d recovery. Insets/boxed regions highlight representative mitochondria and mitochondria–lysosome contact events. (**B**) TEM-based quantification at 0-d recovery: mitochondrial length; number of crista junctions/cristae per 100 mitochondria; number of mitochondria–lysosome contacts normalized to mitochondrial membrane length; and distance between lysosomes and the outer mitochondrial membrane. (**C**) Corresponding TEM-based quantification at 15-d recovery. (**D and F**) Representative confocal images of mitochondria (MitoTracker, red) and lysosomes (LysoTracker, green) at 0-d recovery and 15-d recovery; nuclei stained with Hoechst (blue). (**E and G**) Quantification of lysosome-associated fluorescence readouts reflecting enhanced mitochondria–lysosome association at 0-d recovery and 15-d recovery. Data are shown as mean ± SD. For each recovery time point, statistical significance was assessed using one-way ANOVA followed by Dunnett’s multiple-comparisons test relative to the time-matched control group. *P ≤ 0.05, **P ≤ 0.01, ***P ≤ 0.001.

To validate the structural basis of TFS-induced mitochondrial dysfunction, we performed transmission electron microscopy (TEM) in cells exposed to TFS (2.5 or 25 μg/L) for 14 d and analyzed cells either immediately (0-d recovery) or after 15 d in TFS-free medium (15-d recovery). Control cells displayed elongated mitochondria with intact outer membranes and well-organized cristae, whereas TFS caused a dose-dependent ultrastructural collapse characterized by mitochondrial shortening/fragmentation, blurred membrane boundaries, and pronounced cristae loss (Fig. 4A). Quantification confirmed significant reductions in mitochondrial length and crista junction/cristae at both doses, with only partial restoration after withdrawal that remained markedly below time-matched controls (Fig. 4B and C). In parallel, TEM revealed that TFS increased lysosome abundance and mitochondria–lysosome coupling, with lysosomes frequently juxtaposed to or partially enveloping damaged mitochondria; morphometric analyses showed increased mitochondria–lysosome contacts (normalized to mitochondrial membrane length/surface) and decreased lysosome-to-outer mitochondrial membrane distance, changes that persisted after 15-d recovery, suggesting a stable shift toward enhanced organelle coupling.

We corroborated these TEM observations using fluorescence imaging of mitochondria and lysosomes. Co-staining with MitoTracker and LysoTracker revealed increased mitochondria–lysosome association after TFS exposure (Fig. 4D), accompanied by a strong elevation of lysosome-associated fluorescence readouts (Fig. 4E). Although these signals declined after 15-d recovery, they remained significantly higher than controls (Fig. 4F and G), supporting persistent lysosome engagement with mitochondria following TFS withdrawal. Therefore, TFS not only damages mitochondrial ultrastructure but also promotes sustained mitochondria–lysosome coupling, consistent with prolonged activation of mitochondria-directed quality control pathways.

### TFS induces sustained Drp1 upregulation and mitochondrial recruitment to drive mitochondrial fission

**Fig 5:**
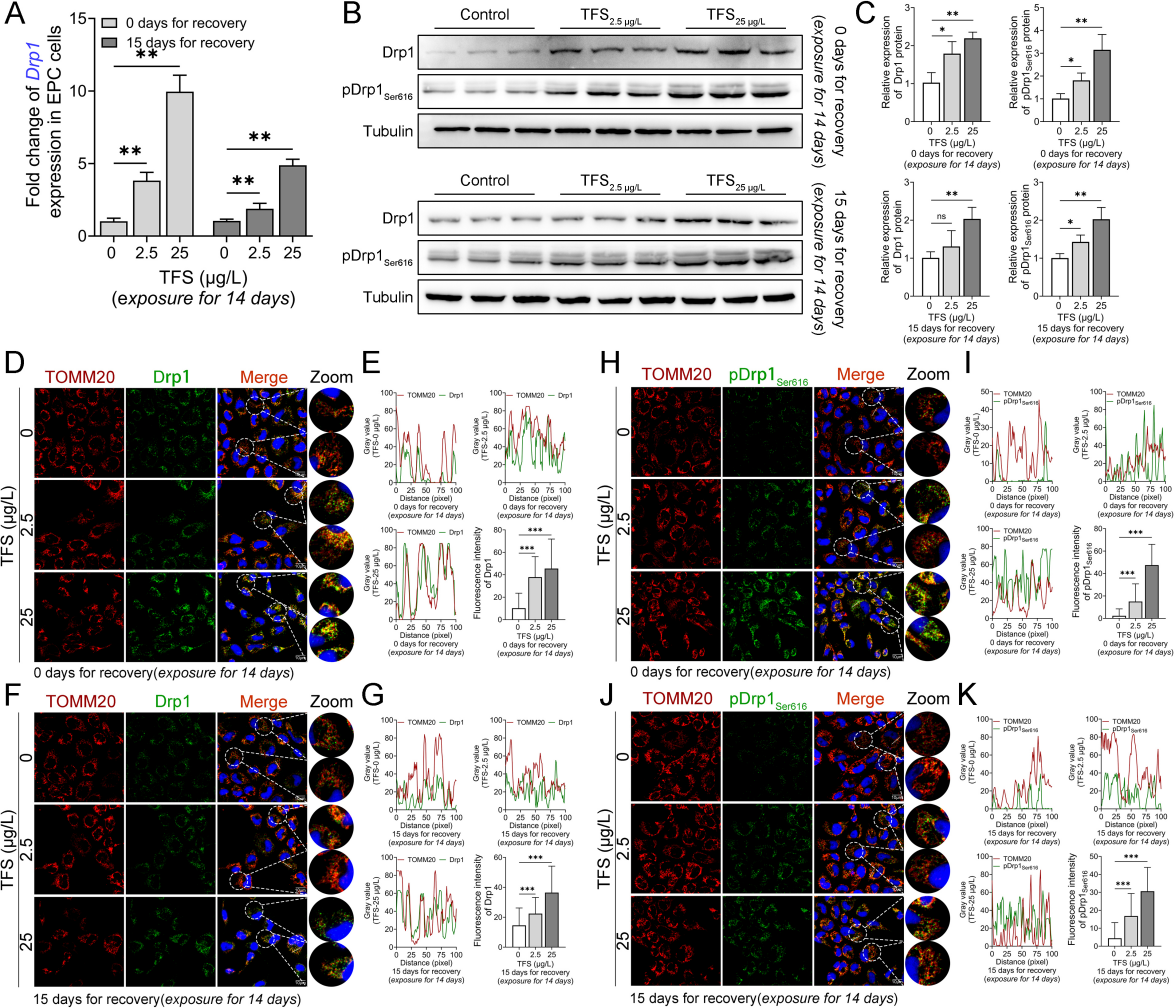
TFS induces sustained Drp1 expression, Ser616 phosphorylation, and mitochondrial recruitment in EPC cells. (**A**) RT-qPCR quantification of *drp1* mRNA expression in EPC cells after 14-d exposure to TFS (0, 2.5, or 25 μg/L), analyzed at 0-d recovery and after 15-d recovery in TFS-free medium. (**B**) Representative immunoblots of Drp1, phosphorylated Drp1 at Ser616 (pDrp1_Ser616_), and Tubulin as a loading control at 0-d recovery and 15-d recovery. (**C**) Densitometric quantification of Drp1 and pDrp1_Ser616_ protein levels normalized to Tubulin. (**D and F**) Confocal images showing TOMM20 (mitochondria, red), Drp1 (green), nuclei (Hoechst, blue), merged images, and zoomed regions at 0-d recovery and 15-d recovery. (**E and G**) Line-scan intensity profiles (TOMM20 vs Drp1) and quantification of Drp1 fluorescence intensity at 0-d recovery and 15-d recovery. (**H and J**) Confocal images showing TOMM20 (red), pDrp1_Ser616_ (green), nuclei (blue), merged images, and zoomed regions at 0-d recovery and 15-d recovery. (**I and K**) Line-scan intensity profiles (TOMM20 vs pDrp1_Ser616_) and quantification of pDrp1_Ser616_ fluorescence intensity at 0-d recovery and 15-d recovery. Data are presented as mean ± SD. For each recovery time point, statistical significance was assessed using one-way ANOVA followed by Dunnett’s multiple-comparisons test relative to the time-matched control group. *P ≤ 0.05, **P ≤ 0.01, ***P ≤ 0.001.

To define the molecular basis of TFS-induced mitochondrial fragmentation, we quantified Drp1 expression, activation, and mitochondrial recruitment in EPC cells exposed to TFS (2.5 or 25 μg/L) for 14 d, followed by either immediate analysis (0-d recovery) or a 15-d recovery in TFS-free medium. Real-time quantitative PCR (RT-qPCR) revealed a robust, dose-dependent induction of *drp1* transcripts after exposure, increasing by 3.76-fold (2.5 μg/L) and 9.79-fold (25 μg/L) relative to controls (*P* < 0.01) (Fig. 5A). Although transcript levels declined after recovery, they remained significantly elevated, indicating incomplete transcriptional normalization. Consistent with the mRNA response, immunoblotting showed that both total Drp1 and its activated form, phosphorylated Drp1 at Ser616 (pDrp1_Ser616_), were markedly increased following TFS exposure (Fig. 5B and C). After 15-d recovery, Drp1 and pDrp1_Ser616_ levels were partially reduced but remained significantly higher than in controls, supporting persistent activation of the fission machinery even after chemical withdrawal.

We next asked whether elevated Drp1 is functionally engaged at mitochondria. Confocal imaging demonstrated that in control cells, TOMM20-labeled mitochondria formed an interconnected network, whereas Drp1 signal was predominantly cytosolic with limited overlap (Fig. 5D). In TFS-exposed cells, Drp1 fluorescence intensity increased, and Drp1 became strongly enriched on TOMM20-positive mitochondria, consistent with enhanced recruitment to the outer mitochondrial membrane (Fig. 5E). Quantification confirmed a significant, dose-dependent increase in Drp1 signal (to 3.70- and 4.45-fold of control at 2.5 and 25 μg/L, respectively; *P* < 0.001), together with elevated TOMM20–Drp1 colocalization. After 15-d recovery, Drp1 intensity and mitochondrial association decreased but remained significantly above control levels (Fig. 5F and G), indicating partial reversibility yet sustained mitochondrial engagement. A similar pattern was observed for pDrp1_Ser616_, which showed persistent enrichment on mitochondria and elevated fluorescence intensity both immediately after exposure and following recovery (Fig. 5H through K). To determine whether persistent Drp1 activation functionally contributes to the heightened viral susceptibility induced by TFS, we next examined the effect of pharmacological Drp1 inhibition using the selective Drp1 GTPase inhibitor Drpitor1a. Drpitor1a did not measurably affect EPC cell viability across the tested concentration range (1.5–100 μM) after 48 or 96 h of treatment (Fig. S6A). Based on these viability data and prior literature, 1 μM was selected for subsequent experiments. Under these conditions, Drpitor1a markedly suppressed SVCV N expression in TFS-exposed cells, largely reversing the increase in viral permissiveness caused by 14-d TFS exposure (Fig. S6B). Together, these data support a functional role for Drp1-dependent mitochondrial dynamics in the enhanced SVCV susceptibility induced by TFS.

### TFS elicits persistent mitophagy activation that incompletely resolves during recovery

**Fig 6:**
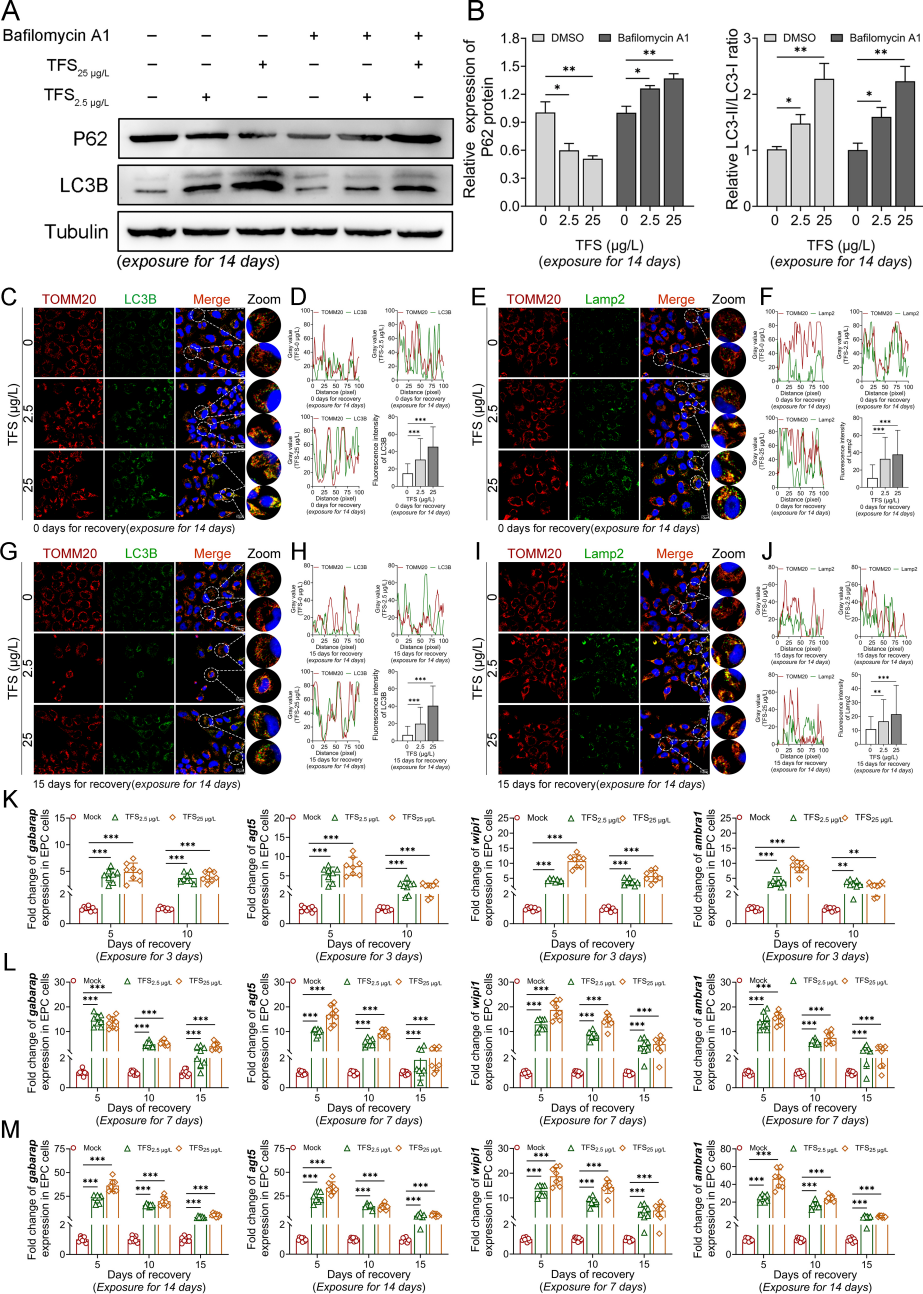
TFS enhances autophagic flux, sustains mitochondria-associated LC3B/LAMP2 signaling, and maintains autophagy-related gene expression during recovery. (**A**) Representative immunoblots of p62, LC3B, and Tubulin in cells exposed to TFS (0, 2.5, and 25 μg/L) for 14 days, with or without Bafilomycin A1 (1 nM) treatment. (**B**) Densitometric quantification of p62 protein abundance and the LC3-II/LC3-I ratio is shown in panel **A**. (**C and G**) Representative confocal images of TOMM20 (mitochondria, red) and LC3B (autophagosome marker, green) with nuclear staining (blue) at 0-d recovery and 15-d recovery; merged and zoomed regions are shown. (**D and H**) Line-scan intensity profiles (TOMM20 vs LC3B) and quantification of LC3B fluorescence intensity (and/or TOMM20–LC3B association, as plotted) at 0-d recovery and 15-d recovery. (**E and I**) Representative confocal images of TOMM20 (red) and LAMP2 (green) at 0-d recovery and 15-d recovery; merged and zoomed regions are shown. (**F and J**) Line-scan intensity profiles (TOMM20 vs LAMP2) and quantification of LAMP2 fluorescence intensity (and/or TOMM20–LAMP2 association, as plotted) at 0-d recovery and 15-d recovery. (**K–M**) RT-qPCR analysis of autophagy/mitophagy-related genes (*gabarap*, *atg5*, *wipi1*, and *ambra1*) in EPC cells during recovery following 3-d, 7-d, or 14-d TFS exposure (0, 2.5, or 25 μg/L). Data are presented as mean ± SD. For panels A and B, statistical significance was assessed using two-way ANOVA followed by an appropriate multiple-comparisons test. For panels D, F, H, and J, statistical significance was assessed within each recovery time point using one-way ANOVA followed by Dunnett’s multiple-comparisons test relative to the time-matched control group. For panels K to M, statistical significance was assessed using two-way ANOVA for the effects of TFS treatment and recovery time, followed by an appropriate post hoc multiple-comparisons test. *P ≤ 0.05, **P ≤ 0.01, and ***P ≤ 0.001.

To determine whether TFS-induced mitochondrial injury is accompanied by activation of mitochondria-directed autophagy, we first examined autophagic flux by assessing microtubule-associated protein 1 light chain 3B (LC3B) and sequestosome 1 (SQSTM1/p62) in EPC cells exposed to TFS (2.5 or 25 μg/L) for 14 d, and used Bafilomycin A1 (1 nM) to assess whether the degradative phase of autophagy was impaired. Under basal conditions, TFS decreased p62 abundance and increased the LC3-II/LC3-I ratio. Following Bafilomycin A1 treatment, the LC3-II/LC3-I ratio remained elevated while SQSTM1/p62 accumulated in TFS-exposed cells (Fig. 6A and B), indicating that autophagic degradation was not blocked. We next examined the abundance of LC3B and the lysosomal membrane protein lysosomal-associated membrane protein 2 (LAMP2). In control EPC cells, TOMM20 displayed a continuous mitochondrial network, whereas LC3B and LAMP2 signals were weak and showed minimal overlap with TOMM20 (Fig. 6C and E). Following 14-d exposure to TFS (2.5 or 25 μg/L), both LC3B and LAMP2 fluorescence intensities increased markedly and displayed pronounced spatial coupling with TOMM20-positive mitochondria. Quantification confirmed significant, dose-dependent elevations (LC3B: 3.70- and 4.45-fold; LAMP2: 3.02- and 3.49-fold at 2.5 and 25 μg/L, respectively; *P* < 0.001), together with enhanced TOMM20–LC3B and TOMM20–LAMP2 colocalization (Fig. 6D and F), consistent with increased autophagosome/lysosome engagement of mitochondria.

We next assessed reversibility after chemical withdrawal. After 15-d recovery in TFS-free medium, LC3B and LAMP2 signals declined compared with the 0-d recovery condition but remained significantly higher than controls and retained visible mitochondrial association (Fig. 6G through J). Quantitatively, LC3B remained 1.53- and 2.50-fold elevated, and LAMP2 remained 1.51- and 1.98-fold elevated at 2.5 and 25 μg/L, respectively (*P* < 0.001), indicating that TFS induces a persistent mitophagy-primed state that incompletely resolves during recovery (Fig. 6H and J). To test whether this persistent phenotype is supported at the transcriptional level, we quantified representative autophagy/mitophagy regulators across exposure durations and recovery windows. In EPC cells, the transcripts of *gabarap*, *atg5*, *wipi1*, and *ambra1* were robustly induced by TFS and remained significantly elevated throughout recovery following 3-d, 7-d, or 14-d exposure, failing to return to baseline even after extended toxin-free culture (Fig. 6K through M). A similar sustained upregulation pattern was observed in zebrafish (Fig. S7), supporting the notion that TFS drives a stable transcriptional reprogramming of the autophagy pathway. Collectively, these results demonstrate that TFS exposure activates mitophagy and maintains autophagy gene programs during recovery, providing a mechanistic basis for prolonged mitochondrial turnover and delayed restoration of antiviral competence.

## DISCUSSION

Environmental pollution increasingly functions as a disease modifier rather than a standalone toxicant, reshaping host physiology in ways that alter susceptibility to circulating pathogens. In humans, epidemiological syntheses link ambient air pollutants with higher risks of respiratory infections and influenza-like illness, consistent with pollution-driven impairment of mucosal defenses and immune responsiveness (35, 36). In aquatic systems, where chemical stress and pathogen pressure often co-occur, multiple studies directly connect contaminant exposure to increased disease sensitivity: juvenile salmon exposed to complex estuarine contaminant mixtures show higher susceptibility to bacterial infection, and contaminant exposure broadly alters immune function in ways that compromise responses to infectious agents (37). Similar patterns emerge for newer pollutants; for instance, microplastic exposure reduces fish disease resistance, and pollutant co-exposure increases viral susceptibility in fish, reinforcing the idea that contaminants can tip host–pathogen dynamics toward infection (38, 39). In fact, increasing evidence points to associations between pollutant exposure and a greater predisposition of aquatic organisms to circulating pathogens, as well as an increase in disease sensitivity, highlighting the need to treat infection outcomes as ecologically relevant endpoints under realistic multi-stressor scenarios. This view is further supported by recent advances in teleost immunology, which increasingly indicate that environmental stress can reshape host defense capacity and susceptibility to infection (40).

Against this multi-stressor host–pathogen framework, TFS represents a particularly relevant model because it is widely used in rice- and horticulture-dominated landscapes and can enter adjacent waters through runoff, spray drift, and drainage. Field and monitoring evidence indicates that TFS is detectable in surface waters of agricultural catchments (reported maxima on the order of sub-µg/L), while paddy ecosystems can generate particularly high short-term aqueous burdens and sustained exposure to transformation products; notably, TFS acid has been documented to accumulate in paddy water to mg/L levels within days after application, underscoring a realistic potential for prolonged mixture exposure (parent + metabolite) downstream of rice agriculture (41). Consistent with this environmental relevance, multiple studies show that TFS can enter aquatic organisms from the water column and undergo trophic transfer with bioaccumulation, thereby posing risks to aquatic biota and ecosystem integrity (42–44). Across taxa, TFS has been linked to developmental and physiological toxicity, including malformations and cardiotoxic phenotypes in zebrafish embryos, highlighting that environmentally relevant concentrations can disturb fundamental vertebrate biology (45). Importantly, our prior work directly connected TFS to antiviral vulnerability: TFS exposure markedly increased SVCV replication in EPC cells in a time-dependent manner, coincident with increased autophagosome abundance, mitochondrial injury by TEM, LC3B-II accumulation, and reduced mTOR Ser2448 phosphorylation, supporting a working model in which TFS-triggered mitochondrial stress engages autophagy programs that ultimately favor viral success (34). Building on that finding, we asked whether longer-term TFS exposure can persistently erode antiviral immune competence in fish after exposure ends. Using 2.5 and 25 μg/L TFS, the levels that are environmentally plausible and below reported paddy-water peaks (170 μg/L) and far below the mg/L accumulation of TFS acid (46), we show that TFS reprograms mitochondrial quality control in a manner that can be incompletely reversible, thereby sustaining susceptibility to viral challenge across extended recovery windows.

Mechanistically, the tight coupling between TFS and mitochondrial injury is biologically plausible because strobilurins are QoIs designed to bind the Qo site of mitochondrial complex III (cytochrome bc1), thereby blocking electron transfer and constraining ATP synthesis. In non-target animal cells, such complex III interference is expected to impose an immediate bioenergetic bottleneck and favor electron leakage, which promotes mitochondrial ROS production, redox imbalance, and collapse of Δψm, a constellation of primary mitochondrial stress signals that can secondarily drive apoptosis and inflammatory rewiring (47). Consistent with this framework, developmental toxicology studies in zebrafish embryos exposed to TFS report pronounced oxidative stress (increased ROS/MDA with antioxidant system disruption) together with broad innate immune gene perturbations, supporting the idea that TFS can simultaneously engage mitochondrial redox stress and immune-relevant transcriptional programs *in vivo* (21). At the organismal and early-life-stage level, multi-strobilurin comparisons indicate that TFS exposure is associated with reduced Δψm and elevated ROS alongside apoptotic signatures, reinforcing mitochondria as a sensitive liability node for this chemical class (48), and can reshape mitochondria-centered quality-control pathways.

Drp1-dependent mitochondrial fission is a gatekeeping step in mitochondrial quality control. By fragmenting the network, it facilitates segregation and subsequent lysosomal turnover of dysfunctional mitochondrial portions (mitophagy). Environmental stressors that elevate mitochondrial ROS, disturb Ca^2+^ handling, or impose bioenergetic restriction commonly converge on Drp1 activation (often indexed by increased Ser616 phosphorylation), shifting cells toward a “pro-fission/pro-mitophagy” state. This logic is supported by toxicant examples in which Drp1-mediated fission contributes to excessive mitophagy and mitochondrial injury, including paraquat-triggered neuronal damage and mitophagy, which is ameliorated by pharmacological inhibition of Drp1 (49). Similar pDrp1_Ser616_–linked fragmentation signatures are also reported for AgNPs, where ROS-Drp1 coupling drives mitochondrial fragmentation and downstream cellular dysfunction (50). Within this framework, strobilurin fungicides are mechanistically well positioned to engage Drp1-centered mitochondrial remodeling because they inhibit mitochondrial respiration at complex III (cytochrome bc1), a perturbation that can simultaneously constrain ATP supply and promote electron leakage/oxidant pressure—conditions that favor mitochondrial depolarization, fission, and mitophagy-biased adaptation. Consistent with this idea, TFS is reported to induce mitochondrial damage–associated mitophagy in human keratinocytes, with evidence for ROS involvement and engagement of canonical mitophagy regulators (30). Importantly, from a disease-ecology perspective, long-term azoxystrobin exposure has been reported to impair antiviral competence in fish alongside disturbed mitochondrial dynamics characterized by excessive fission, supporting the broader notion that strobilurins can promote mitochondrial dynamics imbalance and immunocompromise (32). Indeed, SVCV also activates autophagy/mitophagy in host cells and uses the pathway to facilitate its own genomic RNA replication and increase viral yield; conversely, suppressing autophagy has been proposed as a strategy to restrict SVCV replication (51, 52). *In vivo*-relevant zebrafish work also supports this directionality: interventions that block autophagy can inhibit SVCV replication (53).

**Fig 7:**
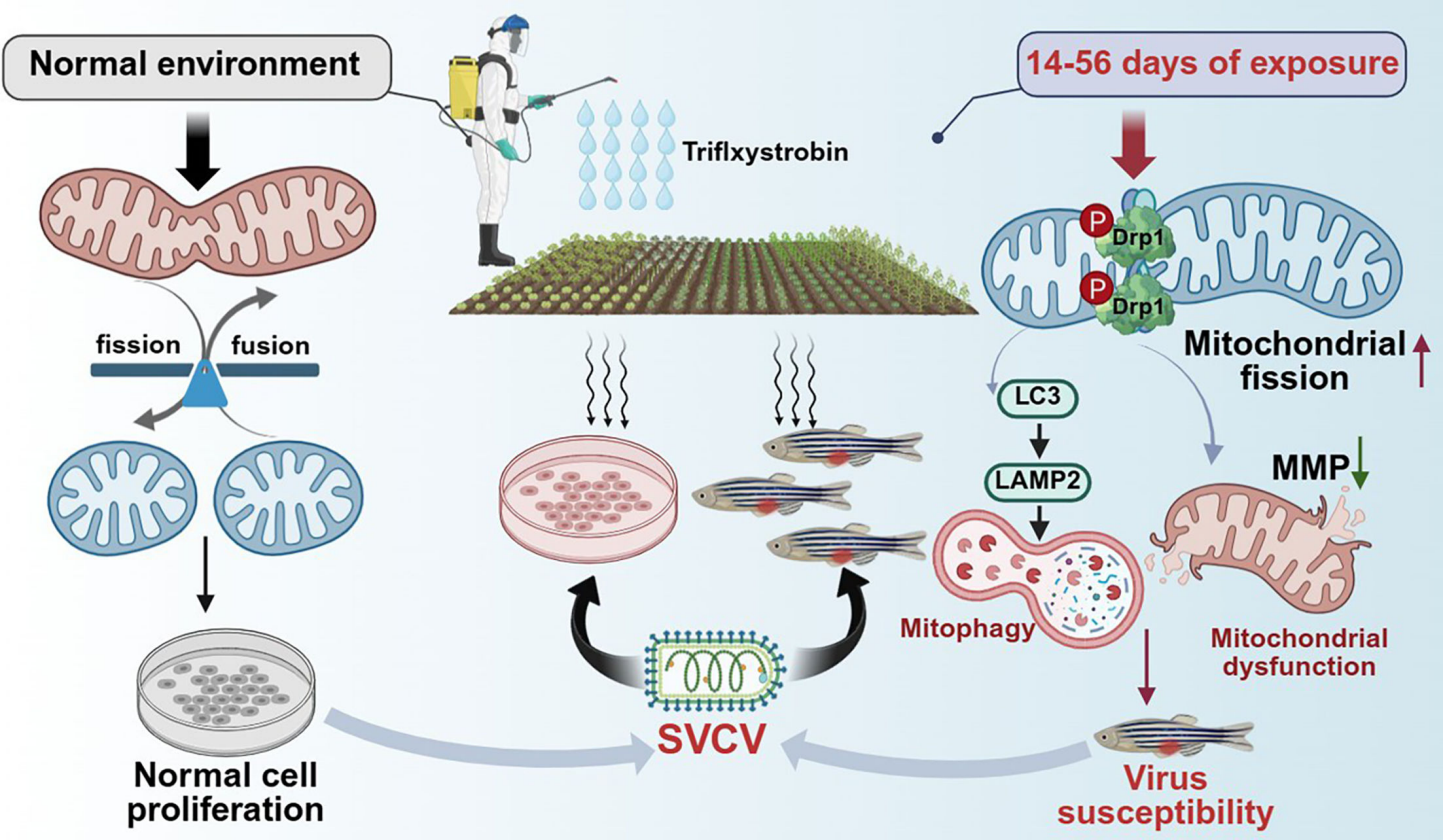
Chronic TFS exposure elevates Drp1/pDrp1_Ser616_, drives Drp1 recruitment to mitochondria, and shifts dynamics toward fragmentation with Δψm loss. Enhanced mitochondria–lysosome contacts and sustained LC3B/LAMP2 signaling indicate persistent mitophagy. This Drp1-dependent mitochondrial “memory” only partially resolves after withdrawal and parallels long-lasting hypersusceptibility to SVCV. Created in BioRender. Lei, L. (2026) https://BioRender.com/tin9qxp.

In summary, our data support a model in which TFS persistently weakens antiviral resistance by driving Drp1-mediated excessive mitophagy. TFS increases Drp1 and pDrp1_Ser616_, promotes Drp1 recruitment to mitochondria, and shifts mitochondrial dynamics toward fragmentation that couples to heightened mitochondria–lysosome engagement and sustained LC3B/LAMP2 signaling. This excessive mitophagy–biased state resolves incompletely after withdrawal and parallels long-lasting SVCV hypersusceptibility during recovery. Together, our findings link a widely used fungicide to durable Drp1-driven mitophagy and underscore long-term immune competence as an ecologically relevant endpoint for aquatic risk assessment (Fig. 7).

## MATERIALS AND METHODS

### Chemical reagent

Analytical-grade TFS (CAS No. 131860-33-8; 100% purity) was purchased from Sigma-Aldrich (St. Louis, MO, USA). A TFS stock solution (500 mg/mL) was prepared in dimethyl sulfoxide (DMSO; Aladdin, Shanghai, China) and stored in the dark at 4°C. Working solutions were freshly prepared immediately before each experiment by diluting the stock into the corresponding cell culture medium or dilution water. To ensure complete dispersion, the appropriate volume of stock was added directly to dilution water under gentle mixing and sonicated for 10 min prior to use. The percent deviations from the nominal concentration were less than 10%, which was considered relatively stable. Unless otherwise indicated, the final DMSO concentration was matched across treatment groups (including vehicle controls) and kept at a level not affecting cell viability or experimental readouts.

### Cell culture, virus propagation, and zebrafish husbandry

EPC cells were kindly provided by Prof. Yong Zhou (Yangtze River Fisheries Research Institute, Chinese Academy of Fishery Sciences) and maintained in Medium 199 (M199; Gibco, USA) supplemented with 10% (vol/vol) fetal bovine serum (FBS) and 1% penicillin–streptomycin at 25°C in a humidified incubator with 5% CO_2_.

The laboratory strain SVCV-0504 (originally isolated from common carp) was propagated in EPC cells. Briefly, EPC monolayers (~80% confluence) were inoculated with SVCV and allowed to adsorb for 1 h at 25°C with gentle rocking. The inoculum was then removed and replaced with M199 containing 2% FBS. When cytopathic effect (CPE) was complete, culture supernatants were collected and clarified at 3,000 × *g* for 10 min at 4°C to remove cell debris. Virus was purified by sucrose-gradient ultracentrifugation (100,000 × *g*, 2 h, 4°C) as described previously, resuspended in sterile phosphate-buffered saline (PBS), aliquoted, and stored at −80°C. Repeated freeze–thaw cycles were avoided. Infectious titers were determined by the 50% tissue culture infective dose (TCID_50_) assay on EPC cells using the Reed–Muench method, yielding a working stock of 1.63 × 10^8^ TCID_50_/mL.

Adult zebrafish (*D. rerio*; body weight 89.56 ± 19.37 mg; body length 2.54 ± 0.21 cm; mean ± standard deviation [SD]) were obtained from the Qing-Feng Zebrafish Breeding Center (Shanghai, China) and acclimated for 30 d prior to experiments. Fish were maintained in a 400-L indoor recirculating system with continuous aeration using ISO standard dilution water (ISO 7346/3-1998) supplied from a sterile circulating water system. The photoperiod was set to 12-h light/12-h dark, and fish were fed commercial dry bloodworm pellets (Yee, Jinan, China) three times daily (08:00, 12:00, and 19:00) at 0.1% body weight per feeding. Only clinically healthy fish with normal appearance and swimming behavior were used. Because the SVCV challenge is temperature-dependent, fish were transferred from standard housing conditions to 18°C water and acclimated for 7 d prior to infection. During SVCV challenge experiments, fish were maintained at 18 ± 1°C throughout the infection period unless otherwise indicated. Fish maintenance and SVCV challenge procedures followed established protocols described previously (32–34).

### TFS exposure in EPC cells and post-exposure recovery

EPC cells were seeded into 12-well plates at 0.5 × 10^6^ cells per well and cultured at 25°C until a confluent monolayer formed. Cells were then exposed to TFS at 2.5 or 25 μg/L, with a vehicle control receiving the corresponding concentration of DMSO (final DMSO ≤0.1% [vol/vol]). Each treatment included three biological replicate wells. Exposures were maintained for up to 14 d. To prevent overgrowth and to keep TFS concentrations close to nominal levels, culture medium (M199 supplemented with 5% FBS) was replaced every 24 h with freshly prepared medium containing the same TFS concentration (or vehicle).

For recovery experiments, after the designated exposure period, TFS-containing medium was completely removed and replaced with TFS-free medium (same formulation), and cells were maintained for up to 15 d. Cell samples were collected at 5-d intervals during recovery (days 5, 10, and 15). Total RNA was extracted using TRIzol reagent (Invitrogen) according to the manufacturer’s instructions. To assess persistence of antiviral suppression, TFS-exposed cells from each recovery time point were infected with SVCV for 48-h post-infection (hpi), and intracellular viral burden was quantified by RT-qPCR targeting the SVCV nucleoprotein (N) gene as described previously (32).

### TFS exposure in zebrafish, recovery, and SVCV challenge

Adult zebrafish were randomly assigned to six groups: Mock, SVCV, TFS 2.5 μg/L, TFS 25 μg/L, TFS 2.5 μg/L + SVCV, and TFS 25 μg/L + SVCV. Each group contained three replicate tanks with 60 fish per replicate. Fish were exposed to TFS at nominal concentrations of 2.5 or 25 μg/L for up to 56 d. During exposure, test solutions were renewed every 24 h using freshly prepared TFS in dechlorinated tap water to maintain water quality and minimize deviation from nominal concentrations. After the exposure phase, fish were transferred to TFS-free water and maintained for a recovery period of up to 95 d. At predefined recovery time points, six surviving fish were randomly sampled from each group, anesthetized with MS-222 (3-aminobenzoic acid ethyl ester methanesulfonate; CAS 886-86-2; Sigma-Aldrich), and immediately snap-frozen in liquid nitrogen. Whole-fish homogenates were used for total RNA extraction followed by RT-qPCR analysis of autophagy-related genes. In parallel, at the same recovery time points, additional TFS-exposed fish were challenged with SVCV for 48 hpi, and viral burden was quantified by RT-qPCR measuring SVCV N gene expression. For each infection batch, mock-challenged fish received virus-free medium processed identically.

### Cytotoxicity of Drpitor1a to EPC cells

The cytotoxicity of Drpitor1a and Bafilomycin A1 in EPC cells was evaluated using a Cell Counting Kit-8 (CCK-8) assay (Beyotime, China). EPC cells were seeded into 96-well plates and cultured to form a confluent monolayer. The culture medium was then replaced with fresh medium 199 supplemented with 5% FBS and containing different concentrations of Drpitor1a (1.5–100 μM). Cells treated with DMSO served as the vehicle control, while untreated cells were used as the control group. After incubation for 48 h or 96 h, cell viability was assessed according to the manufacturer’s instructions.

### RNA isolated, cDNA synthesis, and quantitative PCR analysis

Total RNA from EPC cells or zebrafish samples was extracted using TRIzol reagent (Invitrogen) according to the manufacturer’s instructions. Residual genomic DNA was removed, and first-strand cDNA was synthesized using the HiScript II 1st Strand cDNA Synthesis Kit (+gDNA wiper) (Vazyme). RT-qPCR was performed on an ABI StepOnePlus system (Thermo Fisher Scientific) in 15 μL reactions containing ChamQ Universal SYBR qPCR Master Mix (Vazyme), 0.4 μM of each primer, and 10–50 ng cDNA template. Thermocycling conditions were as follows: 95°C for 30 s, followed by 40 cycles of 95°C for 10 s and 60°C for 30 s. A melt-curve analysis was performed at the end of each run to confirm amplification specificity. The relative expression levels of SVCV *nucleoprotein*, *drp1*, *gabarap*, *atg5*, *wipi1*, *ambra1*, *ifn1*, *viperin*, *mx1*, and *isg15* were normalized to *β-actin* or *18S* rRNA and quantified using the 2^−ΔΔCt^ method. Each biological sample was analyzed in technical triplicate, with at least three independent biological replicates per condition. Primer sequences are provided in Table S1.

### Transcriptome sequencing and preprocessing

EPC cells were exposed to TFS (2.5 or 25 μg/L) for 14 d and harvested for RNA sequencing (RNA-seq). Total RNA was extracted using TRIzol reagent (Invitrogen) following the manufacturer’s protocol. RNA quantity and purity were assessed using a NanoDrop 2000 spectrophotometer, and only samples meeting quality requirements were used for library construction. Sequencing libraries were prepared by Oebiotech (Shanghai, China) and sequenced on an Illumina NovaSeq 6000 platform to generate 150-bp paired-end reads. Raw FASTQ files were quality-controlled and preprocessed using fastp to remove adapter sequences and perform quality filtering. Low-quality reads and reads containing excessive ambiguous bases were discarded, yielding high-quality clean reads for downstream alignment and differential expression analyses.

### Δψm detection

Δψm was assessed in EPC cells using the JC-1 probe (Solarbio) according to the manufacturer’s instructions. JC-1 accumulates in polarized mitochondria as red-fluorescent aggregates, whereas Δψm loss favors green-fluorescent monomers. Briefly, cells were washed once with serum-free M199 and incubated with JC-1 working solution (final 2 μM) for 30 min at room temperature in the dark. Cells were then rinsed and maintained in dye-free M199 for immediate analysis. For fluorescence imaging, JC-1 signals were acquired on a Nikon Ni-E fluorescence microscope under identical exposure settings across groups. Red (aggregate) and green (monomer) fluorescence intensities were quantified, and Δψm was expressed as the red/green ratio.

### Mitochondrial morphology analysis

EPC cells were stained with MitoTracker Red CMXRos (Invitrogen; 1 mM stock in DMSO stored at −20°C, protected from light) diluted to 100 nM in serum-free M199. Cells were rinsed once with serum-free M199 and incubated with MitoTracker for 30 min at room temperature in the dark. Nuclei were counterstained with Hoechst 33342 (10 μg/mL, 5 min, dark). Cells were then imaged in dye-free medium using a ZEISS LSM880 confocal microscope with identical acquisition settings within each experiment. Vehicle controls received matched DMSO (≤0.02%, vol/vol) where applicable. Images were processed in ImageJ/Fiji by background subtraction and analyzed using the MiNA plugin to quantify mitochondrial network features, including mean mitochondrial length, branch length, branch count, network size, and the fraction of globular versus tubular mitochondria (defined by pre-set aspect ratio/form factor thresholds applied consistently across groups). For each condition, at least 30 cells were quantified from ≥ 3 independent biological experiments.

### Mitochondria–lysosome contact analysis

Mitochondria–lysosome coupling was assessed in live EPC cells by dual labeling with MitoTracker Red CMXRos and LysoTracker Green. Cells were incubated with MitoTracker (100 nM, 30 min, room temperature, dark) in serum-free M199, rinsed, and subsequently stained with LysoTracker Green (50 nM, 30 min, room temperature, dark) in serum-free M199. After staining, cells were washed and imaged immediately in dye-free medium using a ZEISS LSM880 confocal microscope under identical acquisition settings across groups. The contacts were defined as LysoTracker-positive puncta positioned in close apposition to MitoTracker-labeled mitochondria with overlapping line-scan intensity profiles across the contact interface. Quantification was performed on ≥30 cells per condition from ≥ 3 independent experiments.

### Ultrastructural analysis of TEM

The samples were fixed in 3% glutaraldehyde prepared in 0.1 M phosphate buffer (pH 7.4) at 4°C for 24 h, rinsed three times in the same buffer, and post-fixed in 1% osmium tetroxide (OsO_4_) for 2 h at 25°C. Samples were dehydrated through a graded ethanol series (65%, 70%, 75%, 80%, and 95%; 15 min each), infiltrated, and embedded in Epon-812 resin. Ultrathin sections (70 nm) were cut using an ultramicrotome, contrasted with uranyl acetate and lead citrate, and imaged at 120 kV on a JEOL transmission electron microscope equipped with a MORADA-G2 camera. For ultrastructural quantification, mitochondria morphology and organelle contact parameters were analyzed in ImageJ/Fiji. Mitochondrial length/size and Mitochondria–lysosome contacts were defined and measured according to previously established criteria (54, 55).

### Immunofluorescence staining

EPC cells grown on coverslips were washed twice with complete culture medium and fixed with 4% formaldehyde for 15 min at room temperature. Cells were then washed three times with PBS and permeabilized with 0.2% Triton X-100 for 10 min. After permeabilization, cells were blocked with Lowenthal serum for 1 h at room temperature to reduce nonspecific binding. Coverslips were incubated with primary antibodies overnight at 4°C, including anti-Drp1 (Santa Cruz; sc-101270, 1:300), anti-TOMM20 (Abcam; ab186735, 1:5,000), anti-LC3B (Abcam; EPR18709, 1:500), and anti-LAMP2 (ABclonal; A1961, 1:250). After primary incubation, cells were washed three times in PBST (PBS containing 0.5% Tween-20) with gentle agitation and incubated with species-matched secondary antibodies for 1 h at room temperature, including Alexa Fluor 488–conjugated goat anti-mouse IgG (Beyotime; A0423, 1:500) and Cy3-conjugated goat anti-rabbit IgG (Beyotime; A0516, 1:500). Cells were washed four times with PBST, counterstained with Hoechst (10 µg/mL) for 10 min, and mounted using an anti-fade mounting medium. Images were acquired on a confocal microscope (Zeiss LSM880) using a 63× oil-immersion objective with identical acquisition settings within each experiment described in the previous studies (56, 57).

### Western blotting

Proteins from EPC cells or zebrafish tissues were extracted in radio-immunoprecipitation assay (RIPA) buffer (150 mM NaCl, 1% Triton X-100, 0.1% SDS, 1 mM EDTA, 50 mM Tris-HCl, pH 7.8) supplemented with protease/phosphatase inhibitors, clarified (12,000 × *g*, 10 min, 4°C), and quantified by bicinchoninic acid (BCA). Mitochondrial, cytosolic, or nuclear fractions were prepared by commercial kits (Beyotime), and purity was verified by markers. Equal protein (20–40 µg per lane) was resolved on 10% sodium dodecyl sulfate polyacrylamide gel electrophoresis (SDS-PAGE) and transferred to nitrocellulose membranes in transfer buffer (25 mM Tris, 192 mM glycine, and 20% methanol). Membranes were blocked for 1 h at room temperature in 5% non-fat milk or 3% bovine serum albumin (BSA) in TBST (TBS buffer/150 mM NaCl, 3 mM EDTA, 50 mM Tris-HCl, pH 8.0; +0.1% Tween-20), then incubated overnight at 4°C with primary antibodies diluted in blocking buffer: anti-Drp1 (Catalog No. sc-101270, Santa Cruz Biotechnology, 1:1,000) and anti-phospho-Drp1 (Ser616) (Catalog No. AP0849, ABclonal, 1:1,000), LC3B (Catalog No. sc-271625, Santa Cruz Biotechnology, 1:1,000), and P62 (Catalog No. sc-48402, Santa Cruz Biotechnology, 1:1,000), followed by horseradish peroxidase-conjugated secondary antibodies for 1 h at room temperature. The images were developed using Chemistar High-sig chemiluminescence western blotting substrate using a Tanon 5200 system. Band intensities were quantified in ImageJ with background subtraction and normalized to fraction markers.

### Statistical analysis

All statistical analyses were performed using GraphPad Prism 9 (GraphPad Software, San Diego, CA, USA). Data are presented as mean ± SD unless otherwise stated. For comparisons involving a single factor with more than two groups at the same time point, statistical significance was assessed using one-way ANOVA followed by Dunnett’s multiple-comparisons test. For experiments involving two independent factors, such as TFS treatment and recovery time or TFS treatment and Bafilomycin A1 treatment, statistical significance was assessed using two-way ANOVA followed by the appropriate post hoc multiple-comparisons test, as specified in the corresponding figure legends. When data sets contained missing values or unequal sample sizes, a mixed-effects model was used where appropriate. A two-tailed unpaired Student’s *t* test was used only for predefined comparisons between two groups. Differences were considered statistically significant at *P* < 0.05.

## Data Availability

The authors confirm that the data supporting the findings of this study are available within the article.

## SUPPLEMENTAL MATERIAL

Supplemental figures jvi.00445-26-s0001.docx Fig. S1 to S7.

Table S1 jvi.00445-26-s0002.docx Sequences of primer pairs used for the analysis of gene expression by RT-qPCR.

ASM does not own the copyrights to Supplemental Material that may be linked to, or accessed through, an article. The authors have granted ASM a non-exclusive, world-wide license to publish the Supplemental Material files. Please contact the corresponding author directly for reuse.
