# Supplementary material for: Trifloxystrobin-triggered Drp1 hyperactivation biases mitophagy and imposes long-lasting SVCV susceptibility in teleost
Source: J Virol. 2026 Jun 1;100(6):e00445-26. doi: 10.1128/jvi.00445-26 (PMC13288995; doi:10.1128/jvi.00445-26)
Supplement: Supplemental figures — Fig. S1 to S7. [file jvi.00445-26-s0001.docx]

**FIG. S1.** Short-term TFS exposure transiently elevates SVCV susceptibility and fully normalizes after extended recovery. EPC cells were exposed to TFS (2.5 or 25 μg/L) for 3 d, allowed to recover in TFS-free medium for 5 or 10 d, and then infected with SVCV. Viral susceptibility was assessed by SVCV nucleoprotein (N) expression (fold change relative to the control). A brief exposure increased N expression after 5 d recovery, whereas 10 d recovery restored N expression to control levels, indicating a reversible, transient phenotype under short exposure. Data are expressed as means ± SD, and statistical comparisons were performed using two-tailed unpaired Student’s *t*-test. **p* ≤ 0.05, ***p* ≤ 0.01, ****p* ≤ 0.001.





**FIG. S2.** Zebrafish were exposed to TFS (0, 2.5, or 25 μg/L) for 7 d (A) or 14 d (B), transferred to pesticide-free water for the indicated recovery periods (5–30 d), and then challenged with SVCV. Viral susceptibility was assessed by measuring SVCV nucleoprotein (N) expression in zebrafish and is presented as fold change relative to the corresponding control group (0 μg/L) at each recovery time point. Data are presented as means ± SD. Statistical comparisons were performed using two-tailed unpaired Student’s *t* test. **p* ≤ 0.05, ***p* ≤ 0.01, and ****p* ≤ 0.001.


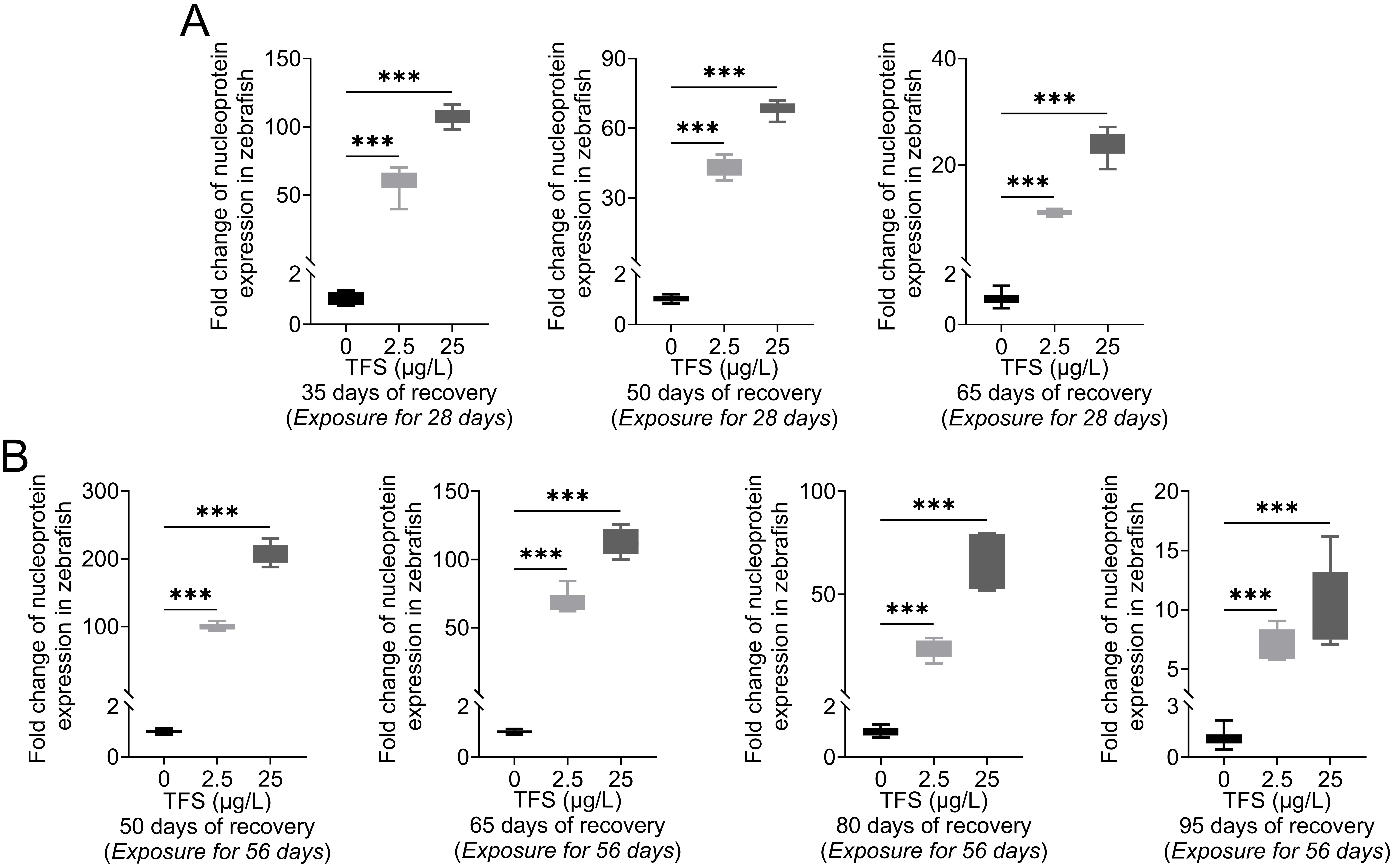


**FIG. S3.** Prolonged TFS exposure induces long-lasting enhancement of SVCV susceptibility in zebrafish. Zebrafish were exposed to TFS (0, 2.5, or 25 μg/L) for 28 d (A) or 56 d (B), transferred to pesticide-free water for the indicated recovery periods, and then challenged with SVCV. Viral susceptibility was evaluated by measuring SVCV nucleoprotein (N) expression in zebrafish and is presented as fold change relative to the corresponding control group (0 μg/L) at each recovery time point. Data are expressed as means ± SD, Statistical comparisons were performed using two-tailed unpaired Student’s *t*-test. **p* ≤ 0.05, ***p* ≤ 0.01, ****p* ≤ 0.001.





**FIG. S4.** Recovery kinetics of interferon-associated antiviral gene expression in EPC cells following TFS exposure. EPC cells were exposed to TFS (0, 2.5, or 25 μg/L) for 3 d (A), 7 d (B), or 14 d (C), and then transferred to TFS-free medium for the indicated recovery periods. Total RNA was extracted at each recovery time point, and the mRNA levels of *ifn1*, *viperin*, *mx1*, and *isg15* were quantified by RT–qPCR. Gene expression is presented as fold change relative to the corresponding time-matched control group. Data are presented as mean ± SD. For each exposure-duration dataset, statistical significance was assessed using two-way ANOVA followed by Dunnett’s multiple-comparisons test, with each TFS-treated group compared with the corresponding control group at the same recovery time point. **p* ≤ 0.05, ***p* ≤ 0.01, ****p* ≤ 0.001.





**FIG. S5.** Recovery kinetics of interferon-associated antiviral gene expression in zebrafish following TFS exposure. Zebrafish were exposed to TFS (0, 2.5, or 25 μg/L) for 7 d (A), 14 d (B), 28 d (C), or 56 d (D), and then transferred to pesticide-free water for the indicated recovery periods (10–95 d, as shown on the x-axes). Total RNA was extracted at each recovery time point, and the mRNA levels of *ifn1*, *viperin*, *mx1*, and *isg15* were quantified by RT–qPCR. Gene expression is presented as fold change relative to the corresponding time-matched control group. Data are presented as mean ± SD. For each exposure-duration dataset, statistical significance was assessed using two-way ANOVA followed by Dunnett’s multiple-comparisons test, with each TFS-treated group compared with the corresponding control group at the same recovery time point. **p* ≤ 0.05, ***p* ≤ 0.01, ****p* ≤ 0.001.


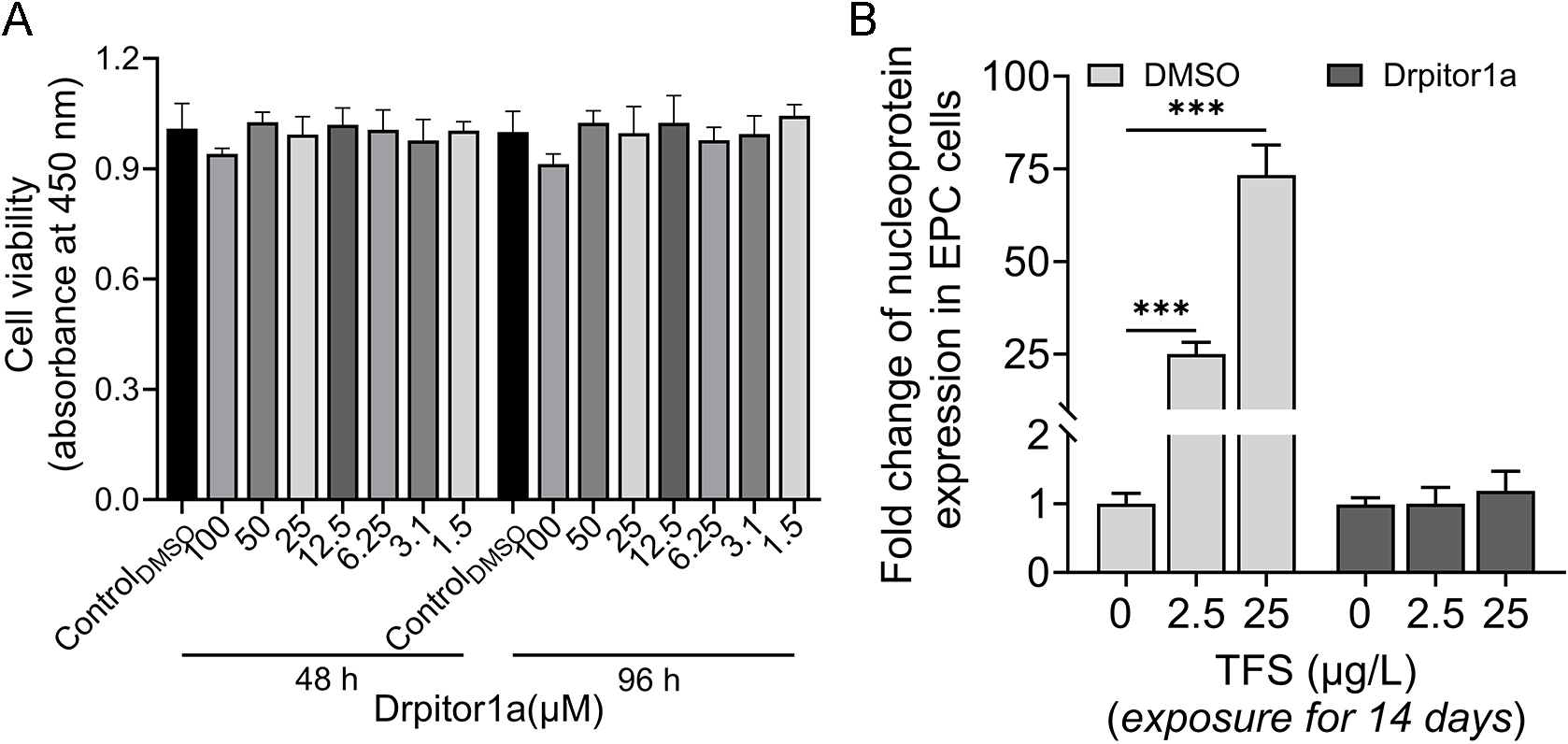


**FIG. S6.** Drpitor1a attenuates TFS-enhanced SVCV permissiveness in EPC cells without detectable cytotoxicity. (A) EPC cells were treated with the indicated concentrations of Drpitor1a for 48 h or 96 h, and cell viability was determined by measuring absorbance at 450 nm. (B) EPC cells were exposed to TFS (0, 2.5, or 25 μg/L) for 14 days, treated with Drpitor1a (1 μM), and then infected with SVCV. Viral susceptibility was assessed by SVCV nucleoprotein (N) expression. Data are presented as mean ± SD. For panel A, statistical significance was assessed within each treatment duration using one-way ANOVA followed by Dunnett’s multiple-comparisons test relative to the DMSO-treated group. For panel B, statistical significance was assessed using two-way ANOVA followed by multiple comparisons between Drpitor1a- and DMSO-treated groups at each TFS concentration. **p* ≤ 0.05, ***p* ≤ 0.01, ****p* ≤ 0.001.





**FIG. S7.** Persistent upregulation of autophagy/mitophagy-related genes in zebrafish during recovery after TFS exposure. Zebrafish were exposed to TFS (0, 2.5, or 25 μg/L) for 7 d (A), 14 d (B), 28 d (C), or 56 d (D) and then transferred to pesticide-free water for the indicated recovery periods (10–95 d, as shown on the x-axes). Total RNA was extracted at each recovery time point and the mRNA levels of *gabarap*, *atg5*, *wipi1*, and *ambra1* were quantified by RT–qPCR. Gene expression is presented as fold change relative to the time-matched control (0 μg/L) group. Data are expressed as means ± SD. For each exposure-duration dataset, statistical significance was assessed using two-way ANOVA followed by Dunnett’s multiple-comparisons test, with each TFS-treated group compared with the corresponding control group at the same recovery time point. **p* ≤ 0.05, ***p* ≤ 0.01, ****p* ≤ 0.001.
