# Supplementary material for: Trifloxystrobin-triggered Drp1 hyperactivation biases mitophagy and imposes long-lasting SVCV susceptibility in teleost
Source: J Virol. 2026 Jun 1;100(6):e00445-26. doi: 10.1128/jvi.00445-26 (PMC13288995; doi:10.1128/jvi.00445-26)
Supplement: Table S1 — Sequences of primer pairs used for the analysis of gene expression by RT-qPCR. [file jvi.00445-26-s0002.docx]

**Table S1** Sequences of primer pairs used for the analysis of gene expression by RT-qPCR.

| Genes |  | Primer sequences (from 5'to 3') |
| --- | --- | --- |
| *β-actin* (EPC cells) | Forward | GCTATGTGGCTCTTGACTTCGA |
|  | Reverse | CCGTCAGGCAGCTCATAGCT |
| *drp1* (EPC cells) | Forward | CAGCGAGGAGAATGGAGTGG |
|  | Reverse | TCGCTGCAGTGACAGCTAAA |
| SVCV *nucleoprotein* (*N*) | Forward | AACAGCGCGTCTTACATGC |
|  | Reverse | CTAAGGCGTAAGCCATCAGC |
| *ambra1* (Zebrafish) | Forward | TCTTTCGAGAAATGGCACCT |
|  | Reverse | CTCTCTGCGTTAGGGACAGG |
| *wipi1* (Zebrafish) | Forward | GTGAGAGGGTAGAGAACAG |
|  | Reverse | GTAACAACGACCCAACATC |
| *atg5* (Zebrafish) | Forward | AGAGAGGCAGAACCCTACTATC |
|  | Reverse | CCTCGTGTTCAAACCACATTTC |
| *gabarap* (Zebrafish) | Forward | GTCTGACCTCACAGTTGGGC |
|  | Reverse | TCCTGGTAGAGCAGTCCCAT |
| *18S* (Zebrafish) | Forward | ACCACCCACAGAATCGAGAAA |
|  | Reverse | GCCTGCGGCTTAATTTGACT |
| *ifn1* (EPC cells) | Forward | CAGAGTCAATGCTCCGCTT |
|  | Reverse | CTCAGATGACTGCCGTTGC |
| *viperin* (EPC cells) | Forward | AGCGAGGCTTACGACTTCTG |
|  | Reverse | GCACCAACTCTCCCAGAAAA |
| *mx1* (EPC cells) | Forward | ATGAATCCTGGAAGCCCTC |
|  | Reverse | GAACTTCGGGAAGAATTTGC |
| *isg15* (EPC cells) | Forward | CAGCCTTGAGGATGATTCCAG |
| *ifn1* (Zebrafish) | Reverse  Forward  Reverse | TGCCGTTGTAAATCAGTCG  GAGCACATGAACTCGGTGAA  TGCGTATCTTGCCACACATT |
| *viperin* (Zebrafish) | Forward | CTGGTGTCGAGAGTATAAGG |
|  | Reverse | CCAACGTACTGGATTGAGAG |
| *mx1* (Zebrafish) | Forward | GACCGTCTCTGATGTGGTTA |
|  | Reverse | GCATGCTTTAGACTCTGGCT |
| *isg15* (Zebrafish) | Forward | AAGTGAGCGGTGATGCTACC |
|  | Reverse | GACTCGAGCTGTCTGCCTTT |
